# Predictive Value of Serum Autotaxin for Hepatocellular Carcinoma Recurrence After Curative Radiofrequency Ablation

**DOI:** 10.1002/cam4.71506

**Published:** 2026-01-07

**Authors:** Takanobu Iwadare, Hiroyuki Kobayashi, Takefumi Kimura, Taiki Okumura, Taro Nakajima, Shun‐ichi Wakabayashi, Yuki Yamashita, Naoyuki Fujimori, Hideo Kunimoto, Satoshi Shimamoto, Koji Igarashi, Takuro Uchida, Takeji Umemura, Naoki Tanaka

**Affiliations:** ^1^ Department of Medicine, Division of Gastroenterology and Hepatology Shinshu University School of Medicine Nagano Japan; ^2^ Consultation Center for Liver Diseases Shinshu University Hospital Nagano Japan; ^3^ Department of Hepatology Shinshu Ueda Medical Center Nagano Japan; ^4^ Department of Hepatology Nagano Municipal Hospital Nagano Nagano Japan; ^5^ Bioscience Division TOSOH Corporation Ayase Kanagawa Japan; ^6^ Division of Travel Medicine and Health, Research Center for GLOBAL and LOCAL Infectious Diseases Oita University Yufu Oita Japan; ^7^ Department of Gastroenterology, Faculty of Medicine Oita University Yufu Oita Japan; ^8^ Shinshu University School of Medicine Nagano Japan; ^9^ Department of Global Medical Research Promotion Shinshu University Graduate School of Medicine Nagano Japan; ^10^ International Relations Office Shinshu University School of Medicine Nagano Japan; ^11^ Research Center for Social Systems Shinshu University Nagano Japan

**Keywords:** autotaxin, biomarker, hepatocellular carcinoma, radiofrequency ablation, recurrence

## Abstract

**Background:**

Despite recent treatment advancements, the high recurrence rate of hepatocellular carcinoma (HCC) following curative therapy remains a significant challenge. Autotaxin (ATX) is a key biomarker in chronic liver disease that has a yet unclarified role in predicting HCC recurrence. This study examined whether precurative radiofrequency ablation (RFA) serum ATX level could serve as a predictor of HCC recurrence after treatment.

**Methods:**

Fifty‐six HCC patients (37 [66%] male; median age: 74 years) treated by curative RFA were retrospectively analyzed.

**Results:**

Twenty‐one patients experienced HCC recurrence during follow‐up. ATX demonstrated superior predictive performance for HCC recurrence after RFA, with an area under the receiver operating characteristic curve of 0.729, sensitivity of 0.857, and specificity of 0.629. Kaplan–Meier analysis revealed that patients with high ATX had a significantly higher incidence of HCC recurrence than those with low ATX (*p* = 0.0006). In univariate analysis, the significant predictors of HCC recurrence included ATX (≥ 1.323 mg/L; hazard ratio [HR]: 6.49; 95% confidence interval [CI]: 1.90–22.13; *p* = 0.003), fibrosis‐4 index (≥ 3.524; HR: 2.75; 95% CI: 1.00–7.55; *p* = 0.050), and mac‐2 binding protein glycosylation isomer (≥ 3.85; HR: 2.12; 95% CI: 1.08–4.19; *p* = 0.030). According to multivariate Cox proportional hazards analysis with seven different models adjusting for age and various established biomarkers, ATX consistently emerged as the only significant independent predictor of HCC recurrence, with HR values ranging from 6.38 to 10.50 (all *p* < 0.05).

**Conclusion:**

Serum ATX is a promising biomarker for predicting HCC recurrence post‐RFA treatment that may outperform conventional markers.

## Graphical abstract

Serum autotaxin (ATX) is a novel and robust biomarker for predicting hepatocellular carcinoma (HCC) recurrence after curative radiofrequency ablation (RFA). Elevated ATX levels independently predict recurrence, outperforming traditional markers and highlighting its potential for personalized post‐RFA surveillance strategies.

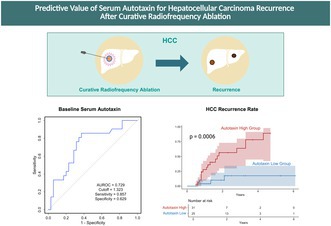

**Abbreviations**
4C7S: type IV collagen 7S
AFP: alpha‐fetoprotein
ALT: alanine aminotransferase
AST: aspartate aminotransferase
ATX: autotaxin
AUROC: area under the receiver operating characteristic curve
BMI: body mass index
CI: confidence interval
CT: computed tomography
ENPP2: ectonucleotide pyrophosphatase/phosphodiesterase family member 2
FIB‐4: fibrosis‐4 index
HA: hyaluronic acid
HBV: hepatitis B virus
HCC: hepatocellular carcinoma
HCV: hepatitis C virus
HR: hazard ratio
IDR: intrahepatic distant recurrence
IQR: interquartile range
LPA: lysophosphatidic acid
LTP: local tumor progression
M2BPGi: mac‐2 binding protein glycosylation isomer
MASLD: metabolic dysfunction‐associated steatotic liver disease
MRI: magnetic resonance imaging
PIVKA‐2: protein induced by vitamin K absence or antagonist‐II
Plt: platelet count
RFA: radiofrequency ablation
ROC: receiver operating characteristic
T‐bil: total bilirubin
ULN: upper limits of normal
VEGFR: vascular endothelial growth factor receptor
γ‐GT: gamma‐glutamyl transferase

## 1 Introduction

Hepatocellular carcinoma (HCC) represents one of the most significant global health challenges in oncology, ranking as the eighth most common cancer and the third leading cause of cancer death worldwide [1]. The disease burden of HCC has increased dramatically over the past three decades, with approximately 747,000 new cases diagnosed and 480,000 deaths recorded in 2019 alone, which is a 70% increase since 1990 [2].

Despite the recent advances in operative techniques and local ablative therapies such as radiofrequency ablation (RFA), which have become a standard curative treatment option for early‐stage HCC, disease recurrence remains a significant clinical challenge that diminishes patient outcomes. Identifying reliable biomarkers to predict HCC recurrence after curative RFA is therefore crucial for improving patient surveillance and management strategies.

First identified in melanoma cell culture studies, autotaxin (ATX) is a crucial enzyme encoded by the ectonucleotide pyrophosphatase/phosphodiesterase family member 2 (ENPP2) gene [3]. This protein plays a vital role in lipid metabolism by converting lysophosphatidylcholine to lysophosphatidic acid (LPA) through its phospholipase activity [4]. Serum ATX level has been demonstrated as a reliable marker for assessing both inflammatory activity and fibrosis progression in various liver conditions, including viral hepatitis, primary biliary cholangitis, and nonalcoholic fatty liver disease [5, 6, 7, 8]. The clinical significance of ATX measurement has been well established in Japan, where its testing has been covered by national health insurance since 2018 for monitoring patients with such chronic hepatitis conditions as metabolic dysfunction–associated steatotic liver disease (MASLD) and primary biliary cholangitis [9, 10].

Despite the validated clinical utility of ATX in liver disease assessment, its value in predicting HCC recurrence after curative RFA remains unexplored. To address this issue, the present study explored the association between pre‐RFA serum ATX level and HCC recurrence following curative RFA treatment.

## 2 Methods

### 2.1 Patients and Clinical Examinations

In this retrospective cohort study, 84 patients with HCC who underwent RFA at Shinshu University Hospital (Matsumoto, Japan) between January 2018 and December 2023 were initially registered. Exclusion criteria included patients with insufficient clinical data, those without available serum samples for analysis, and those who experienced recurrence within 6 months. After applying these criteria, a total of 56 patients were ultimately included in the study. The ethnic background of all patients was Asian. Relevant clinical data, including medical history, laboratory results, radiological findings, and prior treatments, were all collected from the patient's medical records. This study was reviewed and approved by the Institutional Review Board of Shinshu University Hospital (approval number: 3244) and conducted according to the principles of the Declaration of Helsinki. Considering the research design, the IRB ethics committee granted an exemption from obtaining informed consent.

Fibrosis‐4 index (FIB‐4) was calculated according to the following formula: (age [years] × aspartate aminotransferase [AST] [IU/L]) / (platelet count [Plt] [10 [9]/L] × alanine aminotransferase [ALT] [IU/L]^1/2^) [11]. Serum ATX concentrations were determined using a two‐site immunoenzymetric assay with the TOSOH AIA system (TOSOH, Tokyo, Japan). This study employed a solid‐phase antibody produced by a rat anti‐human ATX monoclonal antibody‐producing cell clone, designated as R10.23, along with a labeled antibody using the clone designated as R10.21 [10]. Serum samples were collected after overnight fasting and immediately stored at −30°C.

### 2.2 RFA Procedure

All RFA procedures were performed percutaneously under ultrasound guidance with conscious sedation following local anesthetic administration. The Cool‐Tip RFA System (Covidien, Boulder, CO, USA) was utilized for ablation procedures. Tumor localization was primarily achieved using an ultrasound‐computed tomography (CT)/magnetic resonance imaging (MRI) fusion imaging technique. For lesions with limited visibility on fusion imaging, contrast‐enhanced ultrasonography was additionally employed. In cases where tumors were adjacent to critical organs (including the colon, stomach, or diaphragm), artificial ascites or pleural effusion was introduced as a thermal insulation technique to prevent collateral organ injury. The ablation protocol was designed to achieve complete tumor coverage with a minimum 5‐mm circumferential safety margin in the surrounding nontumorous hepatic parenchyma. All RFA procedures in our study were performed by physicians with at least 5 years of experience, with a total of nine physicians.

### 2.3 Patient Follow‐Up

The start of the follow‐up period was defined as the date of RFA. The end of follow‐up was determined as either the date of the final follow‐up visit or death. Treatment response was assessed using CT or MRI scans performed 4–8 weeks after RFA and subsequently at approximately 3‐month intervals in adherence to the Response Evaluation Criteria in Solid Tumors guidelines, version 1.1. HCC diagnosis was based on imaging characteristics, including arterial hypervascularity and venous or delayed‐phase washout observed on contrast‐enhanced dynamic CT and/or MRI. HCC recurrence was classified into three patterns: local tumor progression (LTP) occurring at the margin of the ablation site and tract, intrahepatic distant recurrence (IDR) developing in nonablated liver segments, and extrahepatic metastasis. Liver function was assessed using the Child–Pugh classification system. The treatment strategy for HCC in each patient was determined based on the Japanese HCC practice guidelines [12].

### 2.4 Statistical Analysis

Clinical data were expressed as the number (percentage) or the median (interquartile range [IQR]). Statistical analyses were performed using R software ver. 4.3.0. The Mann–Whitney U test along with the Chi‐square test or Fisher's exact test (when expected cell frequencies were less than 5) was employed for comparisons between the study groups. Wilcoxon matched‐pairs signed‐rank testing was used for evaluating parameters between pre‐RFA and curative RFA. Diagnostic accuracy was evaluated using the area under the receiver operating characteristic curve (AUROC). We adopted the Youden index to identify cutoff values, with the nearest clinically applicable value to the cutoff considered the optimal threshold for clinical convenience. The Kaplan–Meier method and log‐rank testing were employed to estimate disease progression. The Cox proportional hazards model was adopted to assess univariate and multivariate covariates for HCC recurrence. All statistical tests were two‐tailed and evaluated at the 0.05 level of significance.

## 3 Results

### 3.1 Baseline Characteristics

A total of 56 patients underwent curative RFA treatment, comprising 37 men (66%) and a median age of 74 years (IQR: 68–80) (Table 1). Median body mass index (BMI) was 24.7 kg/m^2^ (IQR: 22.0–26.3). Liver disease etiology varied among the patients and included hepatitis B virus (HBV) (*n* = 6), hepatitis C virus (HCV) (*n* = 14), alcoholic liver disease (*n* = 19), MASLD (*n* = 13), and other causes (*n* = 4). For HBV‐associated HCC patients, five out of six patients were treated with nucleoside/nucleotide analogues and had undetectable HBV‐DNA levels. Regarding HCV‐associated HCC patients, 12 out of 14 patients had achieved sustained virological response. Among alcohol‐related HCC patients, 5 out of 19 patients continued alcohol consumption. Most patients (82.1%) presented with solitary tumors, while 10 patients had multiple tumors. Median tumor size was 18 mm (IQR: 14–26). The patients were divided into two groups [13]. There were 52 patients in the single nodular group and 4 patients in the single nodular with extranodular growth group. In three cases, additional RFA was performed when the safety margin was insufficient. Laboratory data showed preserved liver function (median albumin: 4.0 g/dL; total bilirubin [T‐bil]: 0.85 mg/dL; Plt: 14.3 × 10^4^/μL), advanced liver fibrosis markers (FIB‐4: 4.02; hyaluronic acid [HA]: 179 ng/mL, mac‐2 binding protein glycosylation isomer [M2BPGi]: 2.1), and mildly elevated tumor markers (alpha‐fetoprotein [AFP]: 7.3 ng/mL; protein induced by vitamin K absence or antagonist‐II [PIVKA‐2]: 30 mAU/mL). Median serum ATX level was 1.42 mg/L (IQR: 1.09–1.90).

**TABLE 1:** Clinical features and laboratory data (*n* = 56).

	Median (IQR)/n (%)
Age (years)	74 (68–80)
Male	37 (66)
BMI (kg/m^2^)	24.7 (22.0–26.3)
HBV/HCV/ALD/MASLD/other	6/14/19/13/4
Tumor number (solitary/multiple)	46/10
Tumor size (mm)	18 (14–26)
Tumor nodular type (single nodular type/single nodular with extranodular growth type)	52/4
Laboratory data	
Albumin (g/dL)	4.0 (3.7–4.3)
T‐bil (mg/dL)	0.85 (0.69–1.17)
AST (U/L)	33 (23–49)
ALT (U/L)	28 (17–40)
γ‐GT (U/L)	55 (28–119)
Plt (×10^4^/μL)	14.3 (11.2–18.4)
PT‐INR	1.06 (1.02–1.18)
AFP (ng/mL)	7.3 (4.2–28.7)
PIVKA‐2 (mAU/mL)	30 (18–74)
FIB‐4	4.02 (2.35–5.38)
HA (ng/mL)	179 (110–4451)
4C7S (ng/mL)	6.4 (5.1–8.2)
M2BPGi	2.1 (1.2–4.0)
ATX (mg/L)	1.42 (1.09–1.90)
Child–Pugh score (5–6/7–9)	48/8

Abbreviations: AFP, alpha‐fetoprotein; ALD, alcoholic liver disease; ALT, alanine aminotransferase; AST, aspartate aminotransferase; ATX, autotaxin; BMI, body mass index; FIB‐4, fibrosis‐4 index; γ‐GT, gamma‐glutamyl transferase; HA, hyaluronic acid; HBV, hepatitis B virus; HCV, hepatitis C virus; IQR, interquartile range; MASLD, metabolic dysfunction‐associated steatotic liver disease; M2BPGi, mac‐2 binding protein glycosylation isomer; PIVKA‐2, protein induced by vitamin K absence or antagonist‐II; Plt, platelet count; PT‐INR, prothrombin time‐international normalized ratio; T‐bil, total bilirubin; 4C7S, type IV collagen 7S.

### 3.2 Comparison of HCC Nonrecurrence and Recurrence Groups

A total of 21 patients (37.5%) experienced HCC recurrence during the entire follow‐up period (median: 1.9 years, IQR: 1.2–3.0). Recurrence patterns included 7 cases of LTP and 16 cases of IDR, with two overlapping cases. No cases of distant metastases were observed.

Compared with the nonrecurrence group, the recurrence group demonstrated significantly lower albumin (3.8 vs. 4.1 g/dL, *p* = 0.037) and Plt (12.9 × 10^4^ vs. 15.8 × 10^4^/μL, *p* = 0.024) as well as higher AST (41 vs. 29 U/L, *p* = 0.032) prior to RFA (Table 2). Recurrence patients also displayed higher T‐bil (0.98 vs. 0.74 mg/dL, *p* = 0.011), prothrombin time‐international normalized ratio (1.08 vs. 1.05, *p* = 0.022), AFP (9.2 vs. 4.9 ng/mL, *p* = 0.021), FIB‐4 (4.32 vs. 3.41, *p* = 0.009), HA (321.3 vs. 148.0 ng/mL, *p* = 0.029), M2BPGi (4.0 vs. 1.6, *p* = 0.002), and ATX (1.81 vs. 1.22 mg/L, *p* = 0.001) at this time point. Other variables, including ALT, gamma‐glutamyl transferase (γ‐GT), PIVKA‐2, and type IV collagen 7S (4C7S), showed no significant differences. Demographic factors such as age, sex, BMI, and liver disease etiology were also comparable between the groups.

**TABLE 2:** Comparison of clinical characteristics between nonrecurrence and recurrence patients.

	Nonrecurrence (*n* = 35)	Recurrence (*n* = 21)	*p*
Age (years)	75 (68–82)	72 (68–76)	0.122
Male	24 (68.6)	13 (61.9)	0.200
BMI (kg/m^2^)	24.2 (21.4–26.3)	25.3 (22.4–26.2)	0.206
HBV/HCV/ALD/MASLD/other	1/11/10/11/2	5/3 / 9/2 / 2	0.146
Tumor number (solitary/multiple)	31/4	15/6	0.080
Tumor size (mm)	18 (14–28)	16 (13–25)	0.392
Tumor type (single nodular type/single nodular with extranodular growth type)	32/3	20/1	0.342
Laboratory data			
Albumin (g/dL)	4.1 (3.7–4.3)	3.8 (3.6–4.1)	**0.037**
T‐bil (mg/dL)	0.74 (0.61–0.97)	0.98 (0.84–1.18)	**0.011**
AST (U/L)	29 (22–41)	41 (30–83)	**0.032**
ALT (U/L)	25 (16–40)	30 (20–35)	0.781
γ‐GT (U/L)	55 (28–122)	56 (27–108)	0.848
Plt (×10^4^/μL)	15.8 (11.6–20.3)	12.9 (10.0–16.1)	**0.024**
PT‐INR	1.05 (0.99–1.14)	1.08 (1.05–1.25)	**0.022**
AFP (ng/mL)	4.9 (4.0–19.6)	9.2 (6.5–28.7)	**0.021**
PIVKA‐2 (mAU/mL)	29 (18–60)	39 (19–74)	0.471
FIB‐4	3.41 (1.92–4.48)	4.32 (3.57–6.44)	**0.009**
HA (ng/mL)	148 (110.2–311.2)	321.3 (118–579.5)	**0.029**
4C7S (ng/mL)	6.2 (4.8–8.0)	7.6 (5.7–9.1)	0.437
M2BPGi	1.6 (1.1–2.5)	4.0 (2.2–6.9)	**0.002**
ATX (mg/L)	1.22 (1.03–1.74)	1.81 (1.58–2.41)	**0.001**
Child–Pugh score (5–6/7–9)	31/4	17/4	0.221

*Note:* Bold indicates *p* < 0.05.

Abbreviations: AFP, alpha‐fetoprotein; ALD, alcoholic liver disease; ALT, alanine aminotransferase; AST, aspartate aminotransferase; ATX, autotaxin; BMI, body mass index; FIB‐4, fibrosis‐4 index; γ‐GT, gamma‐glutamyl transferase; HA, hyaluronic acid; HBV, hepatitis B virus; HCV, hepatitis C virus; IQR, interquartile range; MASLD, metabolic dysfunction‐associated steatotic liver disease; M2BPGi, mac‐2 binding protein glycosylation isomer; PIVKA‐2, protein induced by vitamin K absence or antagonist‐II; Plt, platelet count; PT‐INR, prothrombin time‐international normalized ratio; T‐bil, total bilirubin; 4C7S, type IV collagen 7S.

### 3.3 Predictive Ability of Serum ATX for HCC Recurrence

#### 3.3.1 Overall HCC Recurrence

**FIGURE 1:**
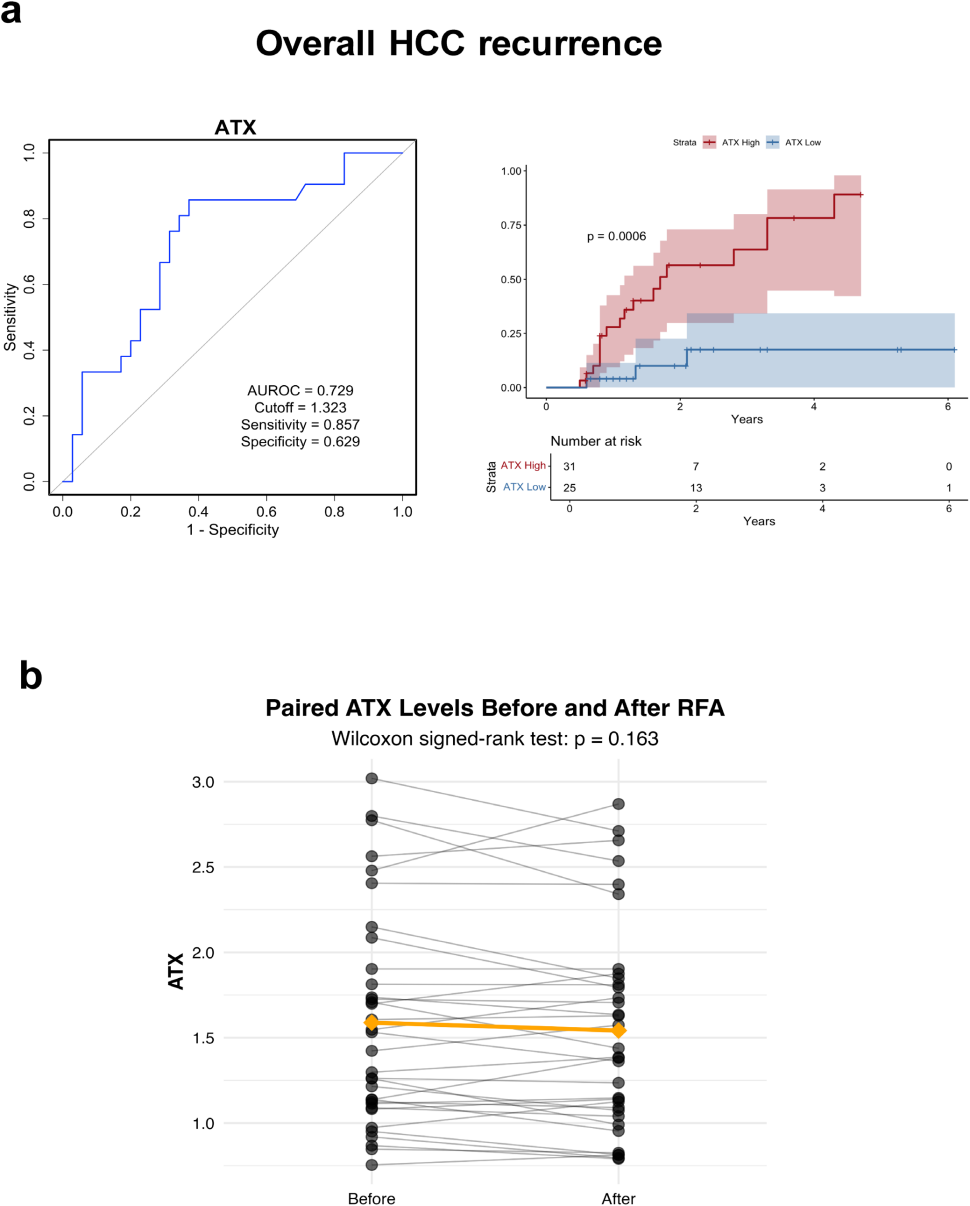
Predictive value of serum ATX for HCC recurrence after RFA. (a) ROC analysis of the predictive performance of serum ATX level for HCC recurrence after RFA on the left and Kaplan–Meier curves of cumulative HCC recurrence rate stratified by high and low ATX levels on the right. (b) Serum ATX levels measured before and after RFA treatment. ATX, autotaxin; AUROC, area under the receiver operating characteristic curve; HCC, hepatocellular carcinoma; RFA, radiofrequency ablation; ROC, receiver operating characteristic.

Receiver operating characteristic (ROC) analysis demonstrated that ATX level prior to RFA had an AUROC of 0.729 for predicting overall HCC recurrence (cutoff: 1.323; sensitivity: 0.857; specificity: 0.629) (Figure 1a). Kaplan–Meier survival analysis revealed a significantly higher cumulative recurrence rate in the high‐ATX group than in the low‐ATX group (log‐rank *p* = 0.0006). To determine an appropriate cutoff value for ATX to identify patients at high risk for recurrence, we initially analyzed the data for the entire cohort and stratified by sex. This approach revealed considerable differences in cutoff values and AUROC between male and female patients (Table S1), likely due to known sex‐based differences in ATX levels [14]. However, applying sex‐specific cutoffs can be cumbersome in clinical practice. To address this issue, we introduced the ATX/ULN ratio, which standardizes ATX levels by dividing by the sex‐specific upper limits of normal (ULN). The ULN for ATX was defined according to the product information provided by the Pharmaceuticals and Medical Devices Agency (PMDA), with reference values of 0.910 mg/L for men and 1.270 mg/L for women [15]. Receiver operating characteristic (ROC) analysis demonstrated that ATX/ULN level prior to RFA had an AUROC of 0.725 for predicting overall HCC recurrence (cutoff: 1.725; sensitivity: 0.619; specificity: 0.8). Kaplan–Meier survival analysis revealed a significantly higher cumulative recurrence rate in the high‐ATX/ULN group than in the low‐ATX/ULN group (log‐rank *p* = 0.0009) (Figure S1a). Notably, the differences in cutoff values and AUROC between sexes were substantially reduced when using ATX/ULN, suggesting that this approach provides a more generalized and practical assessment than absolute ATX values.

#### 3.3.2 Local Tumor Progression (LTP)

**FIGURE 2:**
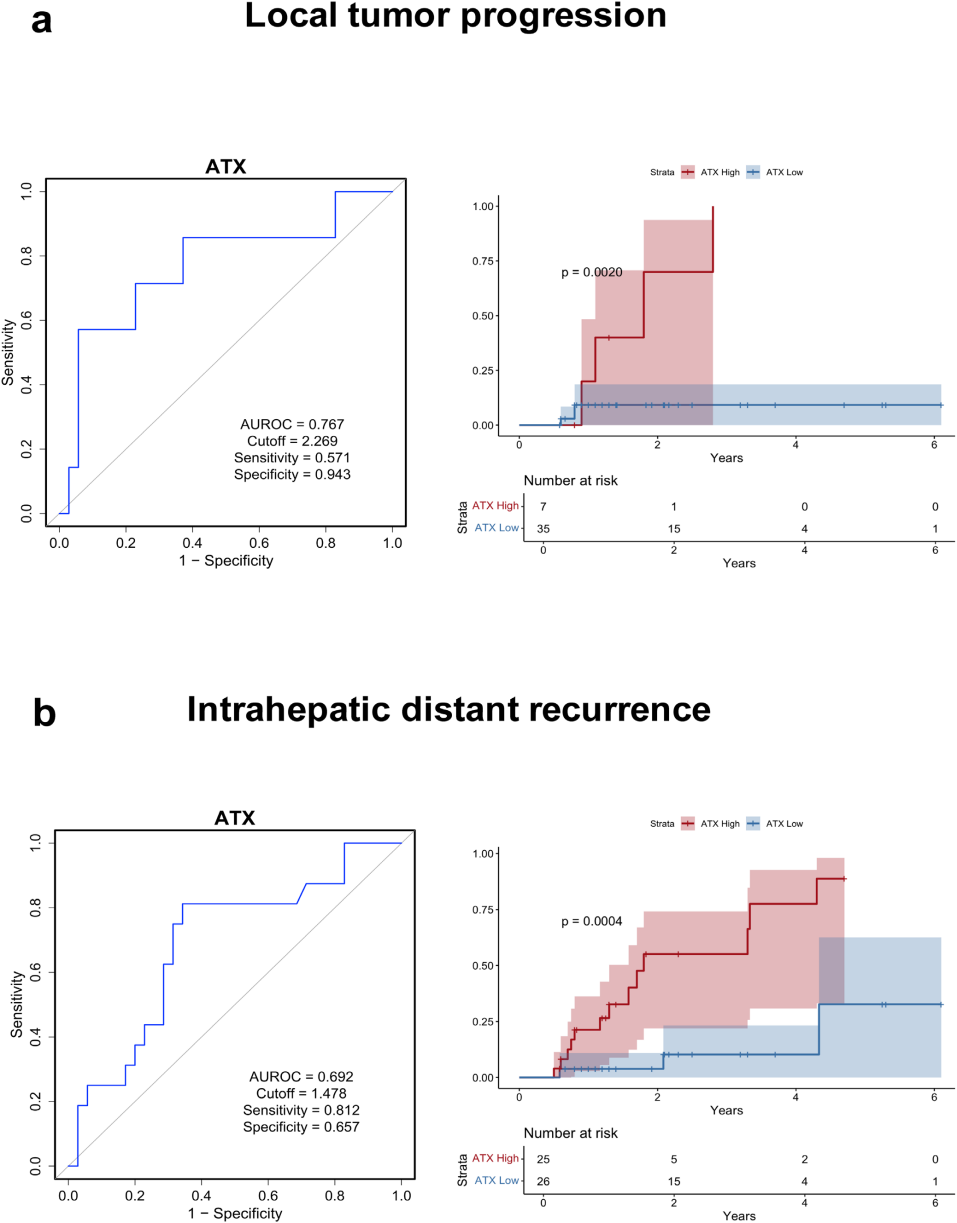
Predictive role of ATX in local tumor progression and intrahepatic distant recurrence of HCC after RFA. Each panel shows ROC analysis on the left and Kaplan–Meier testing stratified by high and low ATX levels on the right. (a) Predictive performance of ATX for local tumor progression of HCC. (b) Predictive performance of ATX for intrahepatic distant recurrence of HCC. ATX, autotaxin; AUROC, area under the receiver operating characteristic curve; HCC, hepatocellular carcinoma; RFA, radiofrequency ablation; ROC, receiver operating characteristic.

ATX showed an AUROC of 0.767 (cutoff: 2.269; sensitivity: 0.571; specificity: 0.943) for predicting LTP (Figure 2a). Kaplan–Meier analysis also indicated a higher LTP rate in the high‐ATX group (log‐rank *p* = 0.0020). ROC analysis demonstrated that ATX/ULN level prior to RFA had an AUROC of 0.62 for predicting LTP (cutoff: 1.4; sensitivity: 0.714; specificity: 0.571). Kaplan–Meier survival analysis demonstrated a trend toward a higher cumulative recurrence rate in the high‐ATX/ULN group compared to the low‐ATX/ULN group, although the difference did not reach statistical significance (log‐rank *p* = 0.1320) (Figure S1b).

#### 3.3.3 Intrahepatic Distant Recurrence (IDR)

We observed an AUROC of 0.692 for ATX predicting IDR (cutoff: 1.478; sensitivity: 0.812; specificity: 0.657) (Figure 2b). Kaplan–Meier analysis showed a significantly higher IDR rate in the high‐ATX group (log‐rank *p* = 0.0004). ROC analysis demonstrated that ATX/ULN level prior to RFA had an AUROC of 0.7 for predicting IDR (cutoff: 1.871; sensitivity: 0.562; specificity: 0.825). Kaplan–Meier survival analysis revealed a significantly higher cumulative recurrence rate in the high‐ATX/ULN group than in the low‐ATX/ULN group (log‐rank *p* = 0.0001) (Figure S1c).

### 3.4 Performance of Various Clinical Scores for Recurrence Prediction

**FIGURE 3:**
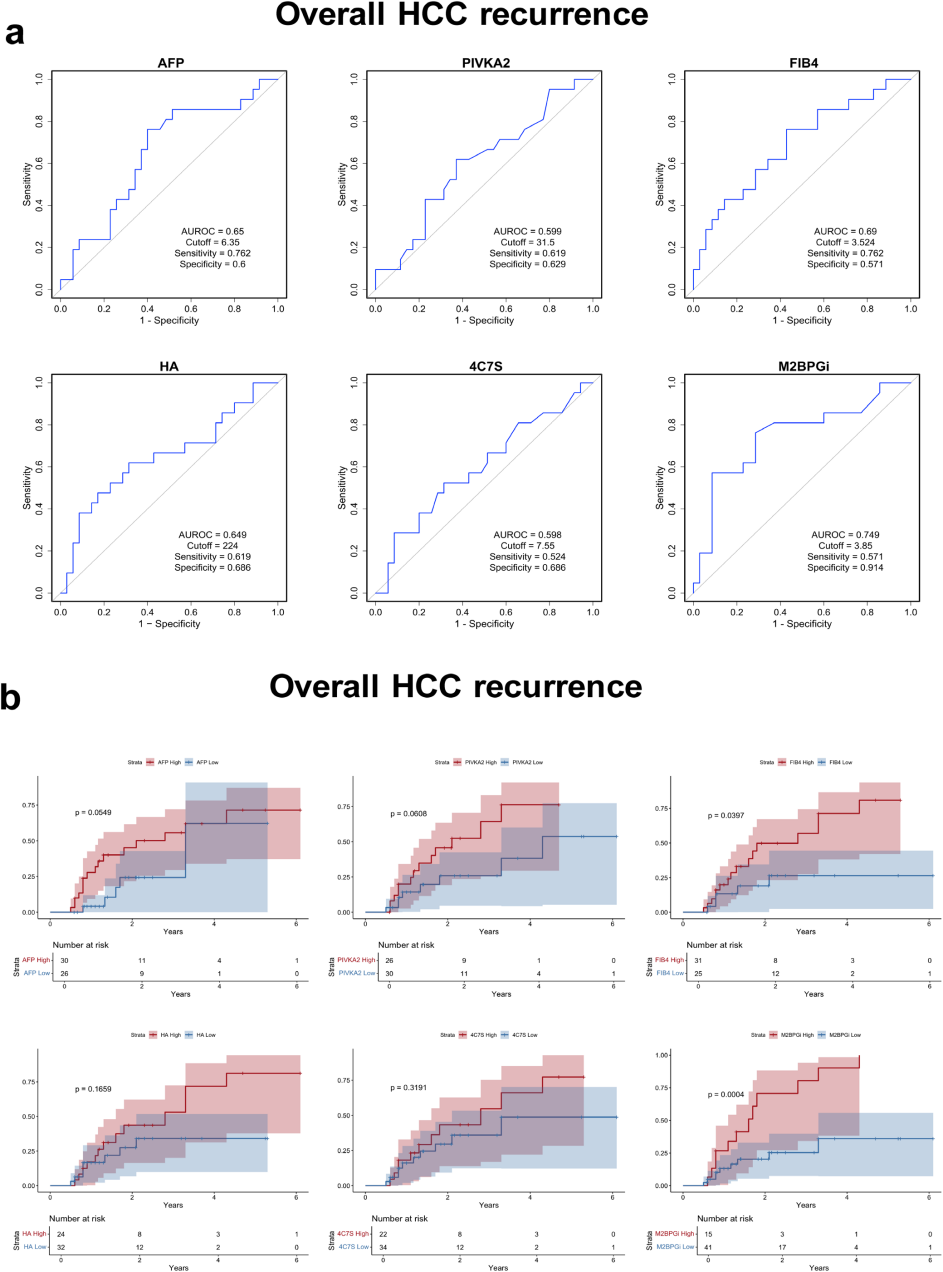
Predictive analysis of conventional biomarkers for HCC recurrence after RFA. (a) ROC analysis illustrating the predictive performance of conventional biomarkers (AFP, PIVKA‐2, FIB‐4, HA, 4C7S, and M2BPGi) for HCC recurrence after RFA. (b) Kaplan–Meier testing of cumulative HCC recurrence rates stratified by high and low levels of each biomarker. AFP, alpha‐fetoprotein; AUROC, area under the receiver operating characteristic curve; FIB‐4, fibrosis‐4 index; HA, hyaluronic acid; HCC, hepatocellular carcinoma; M2BPGi, mac‐2 binding protein glycosylation isomer; PIVKA‐2, protein induced by vitamin K absence‐II; RFA, radiofrequency ablation; ROC, receiver operating characteristic; 4C7S, type IV collagen 7S.

The AUROC values for predicting overall HCC recurrence after RFA were determined for FIB‐4 (0.69), HA (0.649), AFP (0.65), PIVKA‐2 (0.599), 4C7S (0.598), and M2BPGi (0.749) (Figure 3a). At their respective cutoff values, sensitivity and specificity were also calculated for AFP (6.35 ng/mL; sensitivity: 0.762; specificity: 0.6), PIVKA‐2 (31.5 mAU/mL; sensitivity: 0.619; specificity: 0.629), FIB‐4 (3.524; sensitivity: 0.762; specificity: 0.571), HA (224 ng/mL; sensitivity: 0.619; specificity: 0.686), 4C7S (7.55 ng/mL; sensitivity: 0.524; specificity: 0.686), and M2BPGi (3.85; sensitivity: 0.571; specificity: 0.914). Kaplan–Meier analysis revealed significantly higher recurrence rates in patients with elevated liver fibrosis markers, with notable differences in the FIB‐4 high group versus low group (log‐rank *p* = 0.0397) and the M2BPGi high group versus low group (log‐rank *p* = 0.0004) (Figure 3b). No significant differences in recurrence rates were observed between the high and low groups for AFP, PIVKA‐2, HA, or 4C7S.

### 3.5 Changes in Serum ATX Levels After RFA

Analysis of serum ATX values before and after RFA treatment revealed a slightly decreasing trend after treatment, although no statistically significant difference was observed (*p* = 0.163) (Figure 1b). This finding suggested that even after curative HCC treatment with RFA, serum ATX level remained largely unchanged, which potentially reflected the underlying liver condition at the time of carcinogenesis rather than HCC itself.

### 3.6 Univariate and Multivariate Cox Proportional Hazards Models for HCC Recurrence After RFA

In univariate Cox proportional hazards models, the significant predictors of HCC recurrence included ATX (≥ 1.323 mg/mL; HR: 6.49; 95% CI: 1.90–22.13; *p* = 0.003), FIB‐4 (≥ 3.524; HR: 2.75; 95% CI: 1.00–7.55; *p* = 0.050), and M2BPGi (≥ 3.524; HR: 2.12; 95% CI: 1.08–4.19; *p* = 0.030) (Table 3). Although PIVKA‐2 (≥ 31.5 mAU/mL; HR: 2.29; 95% CI: 0.94–5.57; *p* = 0.066) and AFP (≥ 6.35 ng/mL; HR: 2.60; 95% CI: 0.94–5.57; *p* = 0.069) trended toward significance, they did not reach the statistical threshold of *p* < 0.05.

**TABLE 3:** Univariate analysis of factors associated with HCC recurrence after RFA.

	Univariate		
	HR	95% CI of HR	*p*
Age (≥ 65 years)	3.38	0.45–25.30	0.236
Male	0.70	0.29–1.70	0.431
Tumor number (multiple)	1.38	0.53–3.59	0.507
Tumor size (≥ 20 mm)	1.05	0.44–2.50	0.910
AFP (≥ 6.35 ng/mL)	2.60	0.94–5.57	0.069
PIVKA‐2 (≥ 31.5 mAU/mL)	2.29	0.94–5.57	0.066
FIB‐4 (≥ 3.524)	2.75	1.00–7.55	**0.050**
HA (≥ 224 ng/mL)	1.85	0.76–4.47	0.174
4C7S (≥ 7.55 ng/mL)	1.54	0.65–3.64	0.325
M2BPGi (≥ 3.85)	2.12	1.08–4.19	**0.030**
ATX (mg/L) (≥ 1.323 mg/L)	6.49	1.90–22.13	**0.003**

*Note:* Bold indicates *p* < 0.05.

Abbreviations: AFP, alpha‐fetoprotein; ATX, autotaxin; CI, confidence interval; FIB‐4, fibrosis‐4 index; HA, hyaluronic acid; HCC, hepatocellular carcinoma; HR, hazard ratio; M2BPGi, mac‐2 binding protein glycosylation isomer; PIVKA‐2, protein induced by vitamin K absence‐II; RFA, radiofrequency ablation; 4C7S, type IV collagen 7S.

We next performed multivariate Cox proportional hazards analysis to identify potential predictive factors for HCC recurrence after curative RFA. Seven different models were constructed, each including age (> 65 years), ATX level (> 1.323 mg/L), and one each of the following variables: sex, AFP, PIVKA‐2, FIB‐4, HA, M2BPGi, and 4C7S (Table 4). Among all models analyzed (*n* = 56, 21 events), ATX emerged as the only consistently significant independent predictor of HCC recurrence. The HR values for elevated ATX level ranged from 6.38 (95% CI: 1.83–22.31; *p* = 0.004) in the AFP model to 10.50 (95% CI: 2.60–42.49; *p* = 0.001) in the HA model, indicating that patients with high ATX had a substantially increased risk of HCC recurrence. These results indicated that elevated ATX level may serve as a robust independent predictor of HCC recurrence after a complete response to initial RFA treatment, regardless of other clinical and biochemical parameters included in the analysis.

**TABLE 4:** Multivariate analysis for HCC recurrence after RFA.

		HR	95% CI of HR	*p*
Model 1	Age > 65 years	4.94	0.64–38.00	0.125
	Male	1.23	0.50–3.08	0.652
	ATX ≥ 1.323 mg/L	7.64	2.17–26.86	**0.002**
Model 2	Age > 65 years	4.27	0.56–32.45	0.161
	AFP ≥ 6.35 ng/mL	1.63	0.57–4.67	0.363
	ATX ≥ 1.323 mg/L	6.38	1.83–22.31	**0.004**
Model 3	Age > 65 years	4.02	0.51–31.92	0.189
	PIVKA‐2 ≥ 31.5 mAU/mL	1.37	0.54–3.42	0.507
	ATX ≥ 1.323 mg/L	6.65	1.91–23.17	**0.003**
Model 4	Age > 65 years	4.95	0.62–39.50	0.131
	FIB‐4 ≥ 3.524	0.85	0.26–2.80	0.795
	ATX ≥ 1.323 mg/L	7.88	1.90–32.68	**0.004**
Model 5	Age > 65 years	5.66	0.72–44.75	0.100
	HA ≥ 224 ng/mL	0.55	0.20–1.56	0.262
	ATX ≥ 1.323 mg/L	10.50	2.60–42.49	**0.001**
Model 6	Age > 65 years	4.73	0.62–35.83	0.133
	M2BPGi ≥ 3.85	0.90	0.39–2.03	0.792
	ATX ≥ 1.323 mg/L	8.00	1.83–34.95	**0.006**
Model 7	Age > 65 years	4.88	0.64–37.17	0.126
	4C7S ≥ 7.55 ng/mL	0.70	0.28–1.77	0.451
	ATX ≥ 1.323 mg/L	8.58	2.32–31.81	**0.001**

*Note:* Bold indicates *p* < 0.05.

Abbreviations: AFP, alpha‐fetoprotein; ATX, autotaxin; CI, confidence interval; FIB‐4, fibrosis‐4 index; HA, hyaluronic acid; HCC, hepatocellular carcinoma; HR, hazard ratio; M2BPGi, mac‐2 binding protein glycosylation isomer; PIVKA‐2, protein induced by vitamin K absence or antagonist‐II; RFA, radiofrequency ablation; 4C7S, type IV collagen 7S.

## 4 Discussion

### 4.1 Main Findings

This study evaluated the relationship between serum ATX level and HCC recurrence in patients who underwent curative RFA. ROC analysis demonstrated that ATX provided remarkable predictive ability for overall recurrence (AUROC: 0.729), LTP (AUROC: 0.767), and IDR (AUROC: 0.692). In both univariate and multivariate analyses, elevated ATX level (≥ 1.323 mg/L) consistently showed a significant predictive capability for HCC recurrence (univariate HR: 6.49; 95% CI: 1.90–22.13; *p* = 0.003; multivariate HR: 6.38–10.50) independently of conventional tumor markers and liver fibrosis markers.

### 4.2 Context With Published Literature

Several clinical and molecular factors have been identified as predictors of HCC recurrence. Male gender, multiple tumors, and cirrhosis are established independent risk factors for recurrence after RFA for HCC [16, 17, 18]. Traditional tumor markers such as AFP and PIVKA‐2 are also well‐known predictors of IDR, while elevated γ‐GT independently predicts both diminished survival and higher recurrence risk [19] [20]. More recently, a prediction model combining AFP and PIVKA‐2 measurements has emerged as an effective prognosticator of both IDR and extrahepatic metastasis after RFA [21]. Additionally, FIB‐4 score after HCV eradication has been shown to be a useful predictor of IDR [22]. In our study, ATX demonstrated superior predictive performance compared with several of these previously reported markers.

ATX has emerged as a significant contributor to HCC development [23]. By generating LPA and interacting with adhesion molecules such as integrins, ATX facilitates cancer progression and metastasis [24]. Additionally, it has been reported to work in conjunction with vascular endothelial growth factor receptors (VEGFR)‐2 and VEGFR‐3, influencing hepatic vascular development and potentially contributing to HCC onset in patients with chronic hepatitis C [25]. Clinical studies have shown that serum ATX levels can serve as a predictive biomarker for HCC development, particularly following antiviral treatment for hepatitis C [26].

Unlike tumor‐derived markers, elevated serum ATX in HCC patients is believed to reflect background liver pathology rather than direct tumor production [27]. This is supported by findings that ATX levels remain largely unchanged after tumor ablation, show no direct correlation with tumor burden, and exhibit lower expression in tumor tissue compared to surrounding liver tissue. Furthermore, Enooku et al. demonstrated that elevated LPA2 mRNA expression in HCC tissues correlates with poor tumor differentiation and, when combined with high serum ATX levels, serves as a significant risk factor for recurrence [28]. Additionally, high ENPP2 (ATX) expression in HCC has been associated with poor prognosis [29].

Our study further supports these findings, demonstrating that ATX is predictive of both LTP and IDR (AUROC: 0.767 and 0.692, respectively). While LTP is often attributed to technical failure, we minimized this factor by excluding recurrence cases within 6 months. Several mechanisms may explain the association between ATX and LTP. First, ATX may contribute to tumor recurrence through the tumor microenvironment by generating LPA, which is suggested to enhance cell proliferation, angiogenesis, and migration [30]. Second, residual tumor cells after RFA could potentially survive and proliferate over time, particularly in cases with high ATX levels [24]. Third, elevated ATX might reflect higher tumor malignancy, which may be associated with an increased risk of LTP [31].

To further evaluate the clinical utility of ATX, we systematically compared it with both tumor markers (AFP, PIVKA‐2) and fibrosis markers (HA, 4C7S, FIB‐4, M2BPGi) for post‐RFA recurrence prediction. Our AUROC analysis demonstrated that ATX had the second‐highest predictive value among all markers tested, ranking just after M2BPGi. However, in multivariate analysis, ATX emerged as the strongest and most consistent independent predictor of HCC recurrence, with a hazard ratio ranging from 6.38 to 10.50 across different models (*p* values: 0.001–0.006), whereas M2BPGi was not independently associated with recurrence risk. This suggests that M2BPGi may act as a confounding factor for ATX, likely due to their shared association with liver fibrosis. Furthermore, Kaplan–Meier survival analysis revealed that ATX stratified early HCC recurrence risk more effectively than AFP and performed comparably to M2BPGi. Unlike conventional tumor markers, which primarily reflect tumor burden, ATX captures both tumor‐related and microenvironmental factors, including fibrosis‐related recurrence mechanisms, which may explain its superior predictive power [32]. This suggests that ATX is not merely a fibrosis marker but also reflects tumor malignancy potential, making it a more comprehensive biomarker for recurrence prediction.

### 4.3 Strengths and Limitations

The primary strength of this study lies in its demonstration that ATX may be a robust independent predictor of HCC recurrence in patients treated with curative RFA, surpassing the performance of conventional tumor markers, such as AFP and PIVKA‐2. Notably, ATX showed good predictive ability not only for overall recurrence but also for LTP and IDR, with high specificity and sensitivity in ROC analyses.

However, our investigation had several limitations. First, it was conducted as a retrospective study with a relatively small sample size (*n* = 56). The relatively small sample size of our study may limit our ability to detect weaker associations and to simultaneously adjust for multiple confounding factors, particularly in multivariate analysis. Second, since the subjects were uniformly Asian and treated in Japan, where ATX measurement has already been implemented in clinical practice, future research involving larger cohorts from diverse ethnic backgrounds is warranted to confirm our results. Third, due to the limited sample size, we were unable to perform analyses stratified by disease etiology, which could provide important insights into the relationship between ATX levels and HCC recurrence in different underlying liver diseases. Fourth, our findings were not validated in an independent cohort, which will be necessary to establish the generalizability of our results. Finally, the follow‐up period (median: 1.9 years) might not have been sufficient to fully evaluate the long‐term predictive value of ATX for HCC recurrence.

### 4.4 Future Implications

Our findings highlight ATX as a promising biomarker for HCC surveillance. Monitoring serum ATX levels before RFA may aid in predicting recurrence risk, including both LTP and IDR. Given ATX's superior predictive ability over conventional tumor and fibrosis markers, patients with elevated ATX (≥ 1.323 mg/L) may benefit from more intensive post‐RFA surveillance due to their substantially higher recurrence risk (HR: 6.38–10.50). Kaplan–Meier analysis further supports ATX as an effective tool for early recurrence stratification.

To optimize ATX‐based surveillance, further validation in larger, multicenter cohorts is needed. In Japan, where ATX measurement is already part of clinical practice, its integration into surveillance protocols could enhance risk stratification and personalized follow‐up strategies.

## Author Contributions

**Takanobu Iwadare:** conceptualization (lead), project administration (lead), writing – original draft (lead), writing – review and editing (lead). **Hiroyuki Kobayashi:** conceptualization (lead), data curation (equal), project administration (equal), writing – original draft (equal), writing – review and editing (equal). **Takefumi Kimura:** conceptualization (lead), data curation (equal), funding acquisition (equal), project administration (lead), resources (equal), writing – original draft (lead), writing – review and editing (lead). **Taiki Okumura:** data curation (equal), investigation (equal), writing – review and editing (equal). **Taro Nakajima:** data curation (equal), investigation (equal), writing – review and editing (equal). **Shun‐ichi Wakabayashi:** data curation (equal), investigation (equal), software (equal), visualization (equal), writing – review and editing (equal). **Yuki Yamashita:** data curation (equal), investigation (equal), writing – review and editing (equal). **Naoyuki Fujimori:** data curation (equal), investigation (equal), writing – review and editing (equal). **Hideo Kunimoto:** data curation (equal), investigation (equal), writing – review and editing (equal). **Satoshi Shimamoto:** methodology (equal), writing – review and editing (equal). **Koji Igarashi:** methodology (equal), writing – review and editing (equal). **Takuro Uchida:** investigation (equal), writing – review and editing (equal). **Takeji Umemura:** supervision (equal), writing – review and editing (equal). **Naoki Tanaka:** conceptualization (equal), supervision (equal), writing – review and editing (equal).

## Funding

Kimura T, Tanaka N, and Umemura T were supported by a grant from the Japan Agency for Medical Research and Development (AMED) (JP23fk0210125, JP24fk0210125, and JP256f0137007j0001) for conducting this research. This work was also supported by JSPS KAKENHI grants‐in‐aid (22 K20884 and 24 K11087) and the Research Center for GLOBAL and LOCAL Infectious Diseases, Oita University (2024B11).

## Ethics Statement

This study was reviewed and approved by the Institutional Review Board of Shinshu University Hospital (approval number: 3244) and conducted according to the principles of the Declaration of Helsinki.

## Conflicts of Interest

Shimamoto S and Igarashi K are employees of TOSOH Corporation. There is no COI to declare for the other authors.

## Supporting information


**Figure S1:** Predictive role of ATX/ULN in HCC recurrence after RFA.
Each panel shows ROC analysis on the left and Kaplan–Meier testing stratified by high and low ATX/ULN levels on the right. (a) Predictive performance of ATX/ULN for overall HCC recurrence. (b) Predictive performance of ATX/ULN for local tumor progression of HCC. (c) Predictive performance of ATX/ULN for intrahepatic distant recurrence of HCC. ATX, autotaxin; AUROC, area under the receiver operating characteristic curve; HCC, hepatocellular carcinoma; RFA, radiofrequency ablation; ROC, receiver operating characteristic; ULN, upper limits of normal.

**Table S1:** Cutoff and AUROC Values of ATX and ATX/ULN for Predicting HCC Recurrence.

## Data Availability

The data that support the findings of this study are available from the corresponding author upon reasonable request.
