# Supplementary material for: Predictive Value of Serum Autotaxin for Hepatocellular Carcinoma Recurrence After Curative Radiofrequency Ablation
Source: Cancer Med. 2026 Jan 7;15(1):e71506. doi: 10.1002/cam4.71506 (PMC12778304; doi:10.1002/cam4.71506)
Supplement: Supplementary file 1 — Figure S1: Predictive role of ATX/ULN in HCC recurrence after RFA. Each panel shows ROC analysis on the left and Kaplan–Meier testing stratified by high and low ATX/ULN levels on the right. (a) Predictive performance of ATX/ULN for overall HCC recurrence. (b) Predictive performance of ATX/ULN for local tumor progression of HCC. (c) Predictive performance of ATX/ULN for intrahepatic distant recurrence of HCC. ATX, autotaxin; AUROC, area under the receiver operating characteristic curve; HCC, hepatocellular carcinoma; RFA, radiofrequency ablation; ROC, receiver operating characteristic; ULN, upper limits of normal. [file CAM4-15-e71506-s002.pptx]

## Slide 1
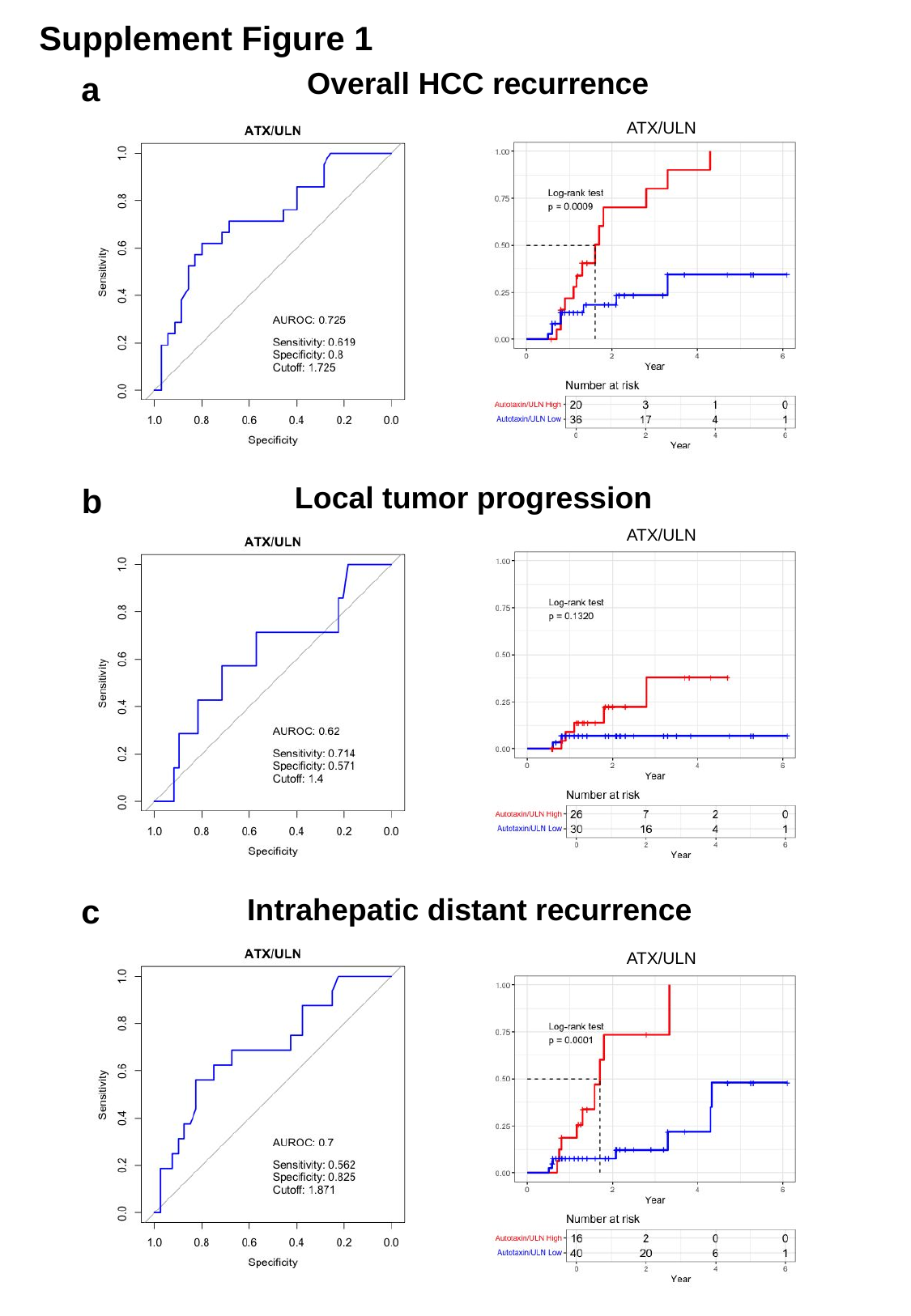

Supplement Figure 1
Overall HCC recurrence
a
ATX/ULN
Local tumor progression
b
ATX/ULN
Intrahepatic distant recurrence
c
ATX/ULN
