# Supplementary material for: Predictive Value of Serum Autotaxin for Hepatocellular Carcinoma Recurrence After Curative Radiofrequency Ablation
Source: Cancer Med. 2026 Jan 7;15(1):e71506. doi: 10.1002/cam4.71506 (PMC12778304; doi:10.1002/cam4.71506)
Supplement: Supplementary file 2 — Table S1: Cutoff and AUROC Values of ATX and ATX/ULN for Predicting HCC Recurrence. [file CAM4-15-e71506-s001.docx]

**Supplement Table1. Cutoff and AUROC Values of ATX and ATX/ULN for Predicting HCC Recurrence**

|  | **Cutoff** | **AUROC** |
| --- | --- | --- |
| **ATX (mg/L)** (Total) | 1.323 | 0.729 |
| **ATX (mg/L)** (Male) | 1.514 | 0.753 |
| **ATX (mg/L)** (Female) | 1.323 | 0.648 |
| **ATX/ULN** (Total) | 1.725 | 0.725 |

ATX, autotaxin; AUROC, area under the receiver operating characteristic curve; HCC, hepatocellular carcinoma; ULN, upper limits of normal.
